# Supplementary figures and images for: Earthworms and plants can decrease soil greenhouse gas emissions by modulating soil moisture fluctuations and soil macroporosity in a mesocosm experiment
Source: PLoS One. 2024 Feb 15;19(2):e0289859. doi: 10.1371/journal.pone.0289859 (PMC10868744; doi:10.1371/journal.pone.0289859)

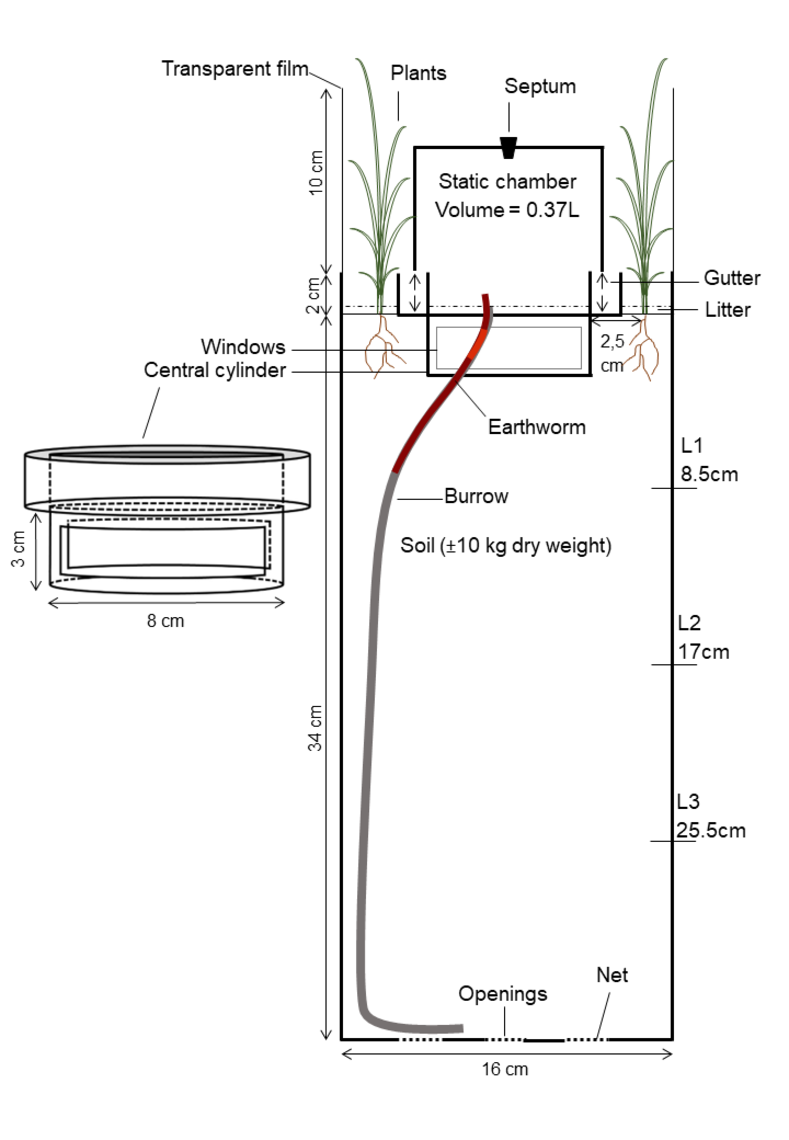

Supplement: S1 Fig — L1 (0–8.5 cm), L2 (8.5–17 cm), L3 (17–25.5 cm) and L4 (25.5–34 cm depth) represent the four different soil layers that were separately analyzed for soil porosity variables. (TIF) [file pone.0289859.s001.tif]

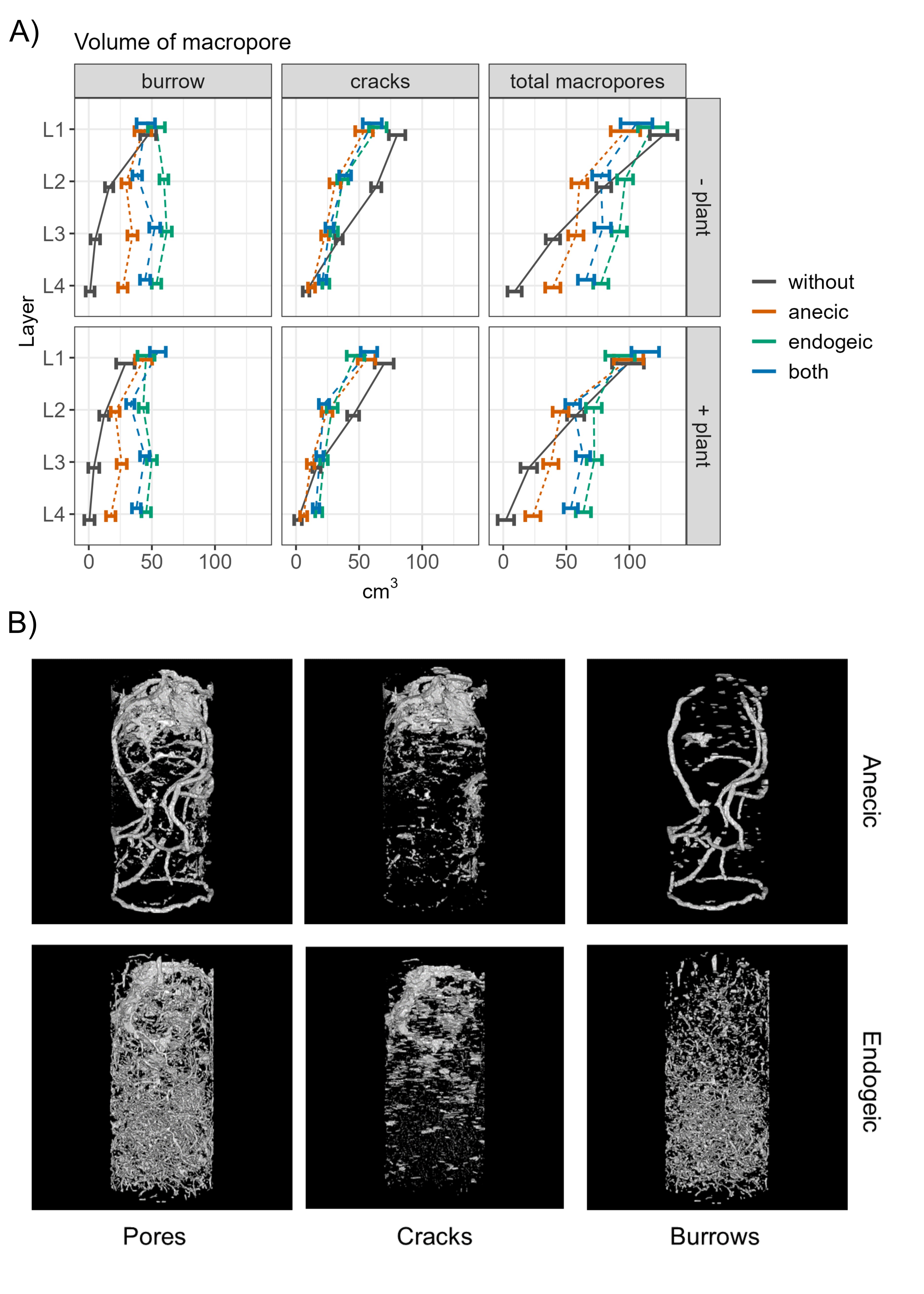

Supplement: S2 Fig — A) The effects of treatments on macroporosity volume differentiated as earthworm burrows, cracks, and total macroporosity (burrows + cracks). Error bars represent ± 1 SEM (L1 for 0–8.5 cm, L2 for 8.5–17 cm, L3 for 17–25.5 cm and L4 for 25.5–34 cm depth; see Fig 1). B) Examples of 3D reconstruction of the soil macroporosity differentiated as burrows, cracks and total (burrows + cracks) for the two earthworm species alone. (TIF) [file pone.0289859.s002.tif]

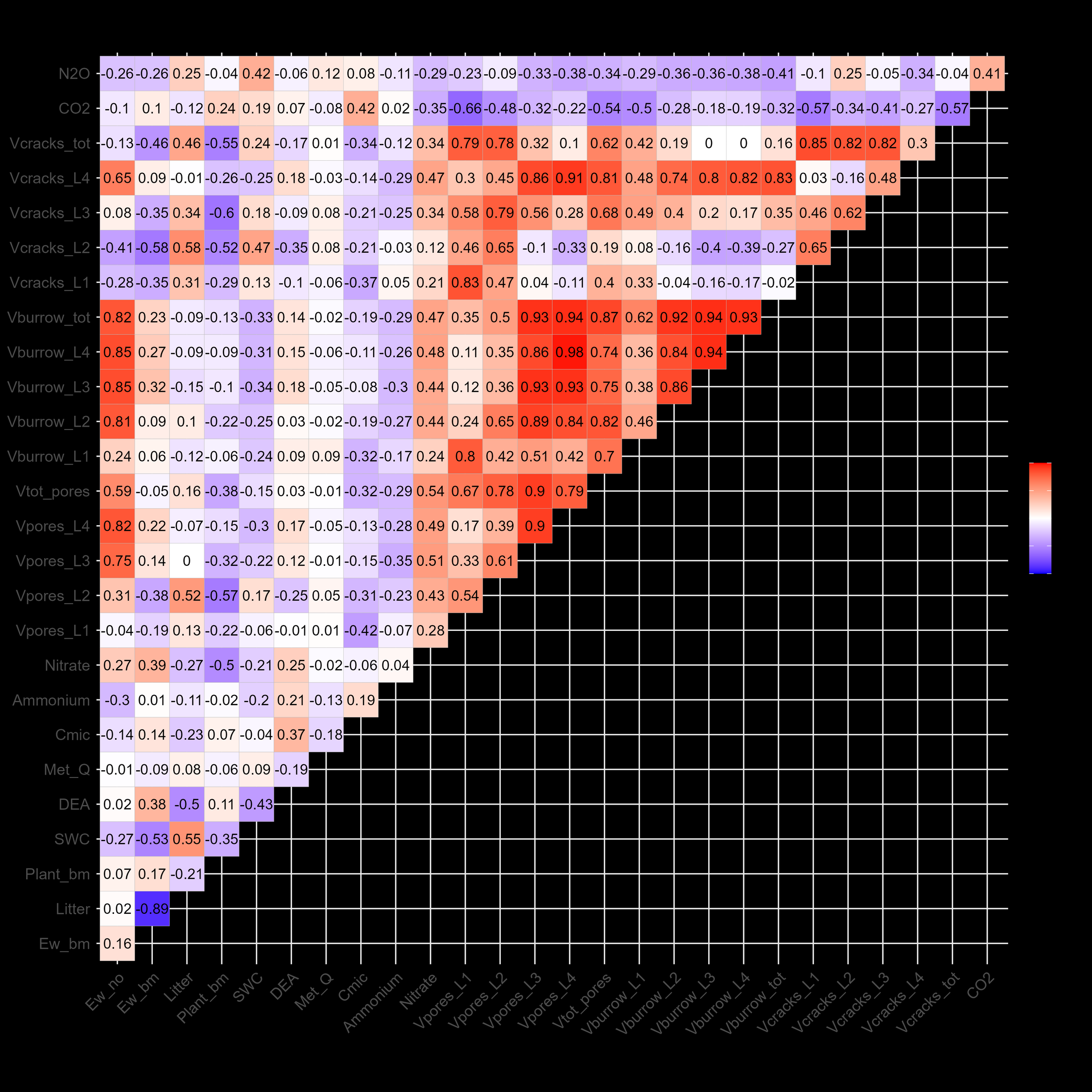

Supplement: S3 Fig — Ew_bm = Earthworm biomass (g FW mesocosm-1), Ew_no = Number of added earthworms per mesocosm (number), SWC = Soil water content relative to field capacity (% of field capacity), Plant_bm = Aboveground plant biomass (g DW mesocosm-1), Litter = Percentage of soil surface covered by litter (%), EA = Potential denitrification enzymatic activity (μg N g-1 soil DW h-1), Cmic = Microbial biomass C (μg Cmic g soil -1 DW), BR = Microbial basal respiration (μg C-CO2 g-1soil DW h-1), Met_Q = Microbial metabolic quotient (μg C–CO2 μg-1 h-1), NH4+ = Ammonium content in soil at the end of the experiment (mg kg-1), NO3- = Nitrate content in soil at the end of the experiment (mg kg-1), Vpores_L1-4 & tot = Macropore (burrow + cracks) volume estimated from CT scan in the 0–8.5 cm layer (L1), 8.5–17 cm layer (L2), 17–25.5 cm layer (L3), 25.5–34 cm layer (L4), and in the whole mesocosm (tot) (cm3), Vburrows_ L1-4 & tot = Burrow volume estimated from CT scan in the 0–8.5 cm layer (L1), 8.5–17 cm layer (L2), 17–25.5 cm layer (L3), 25.5–34 cm layer (L4), and in the whole mesocosm (tot) (cm3), Vcracks_L L1-4 & tot = Cracks volume estimated from CT scan in the 0–8.5 cm layer (L1), 8.5–17 cm layer (L2), 17–25.5 cm layer (L3), 25.5–34 cm layer (L4), and in the whole mesocosm (tot) (cm3). (TIF) [file pone.0289859.s003.tif]

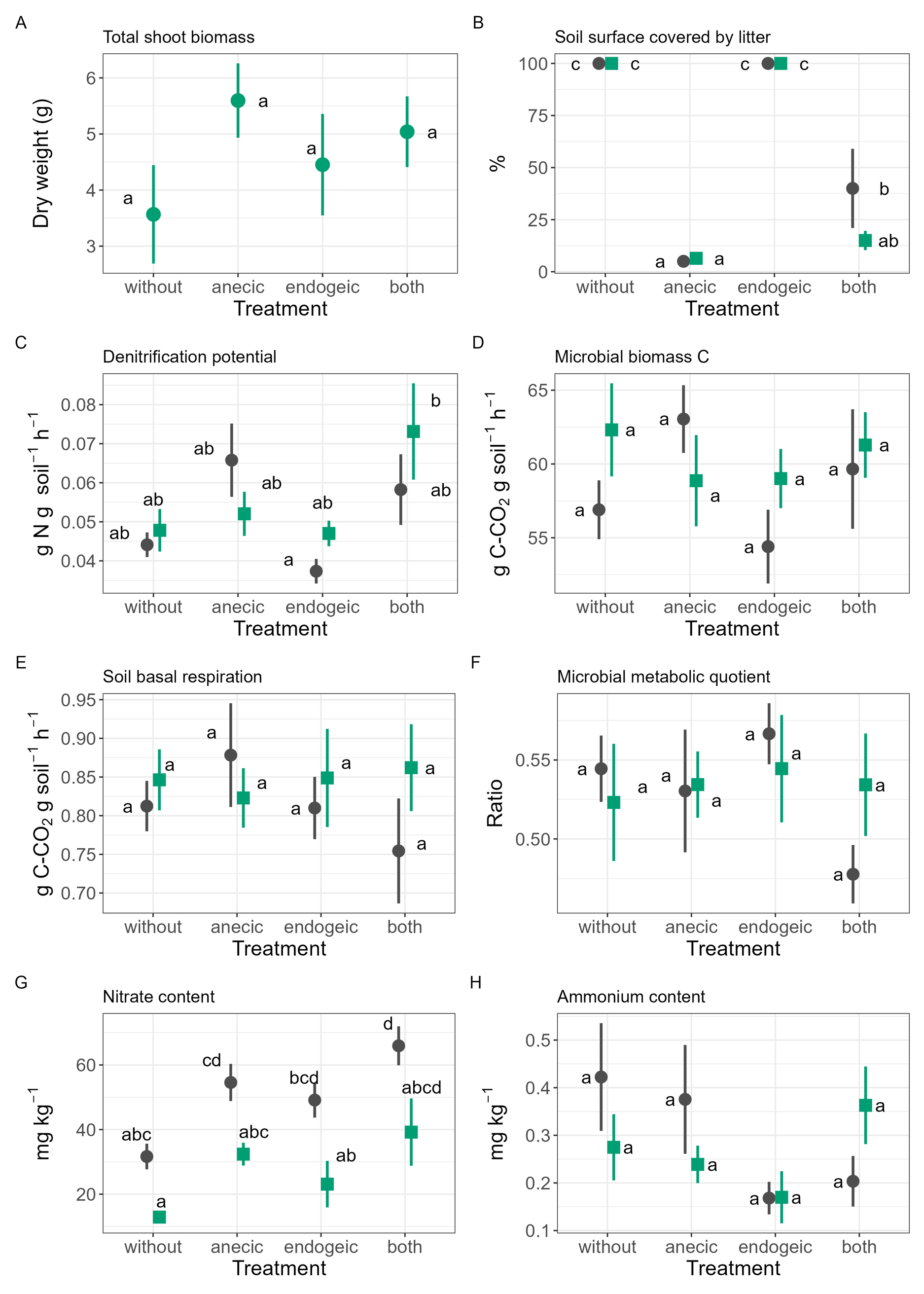

Supplement: S4 Fig — Green = in presence of plant, dark grey = in absence of plant. Different letters represent significantly different treatments according to Tukey’s HSD post hoc test. Error bars represent ± 1 SEM. (TIF) [file pone.0289859.s004.tif]

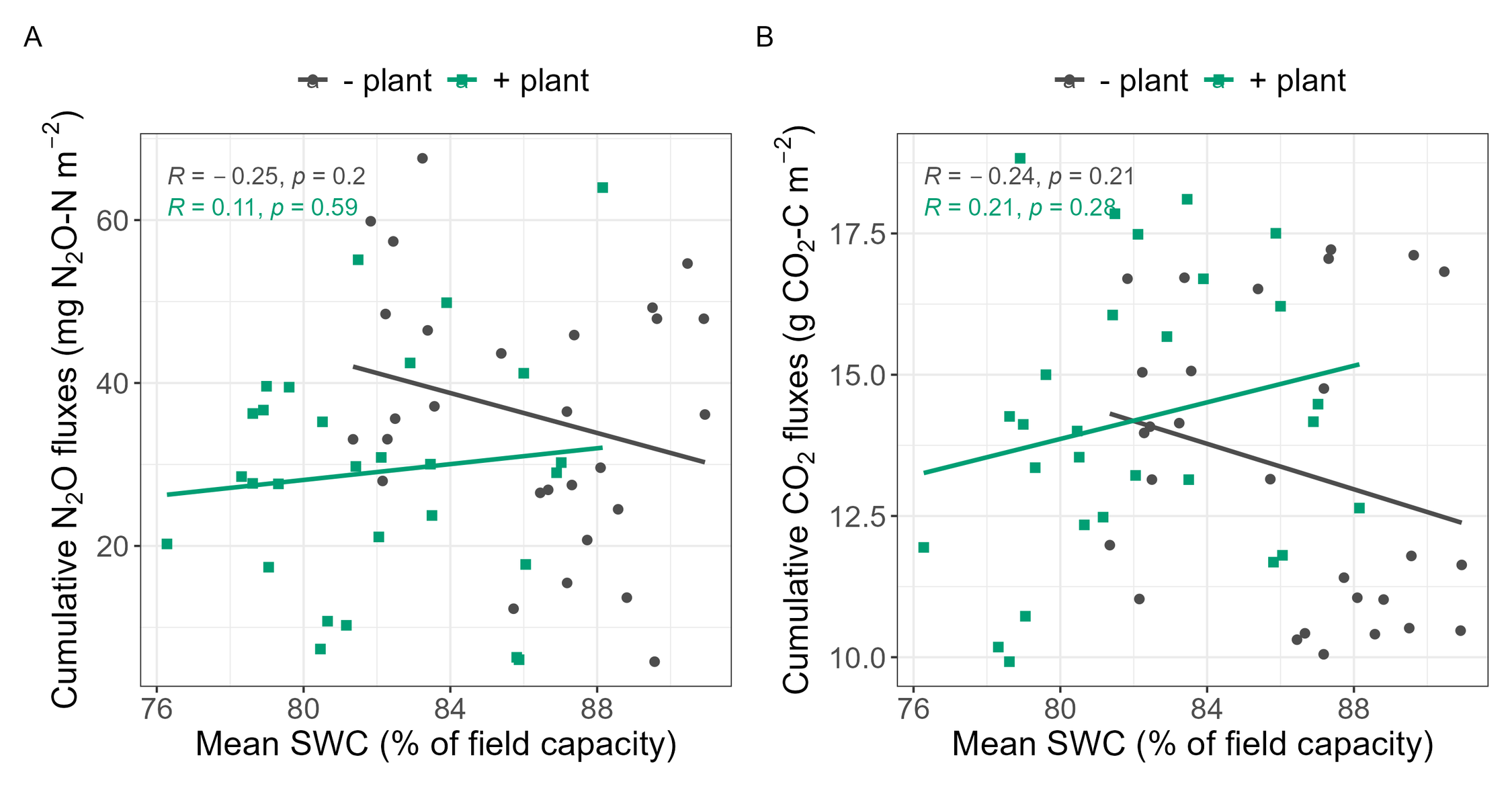

Supplement: S5 Fig — Cumulative N2O (A) and CO2 (B) emissions as affected by Plant×SWC interaction, where SWC represents the 3-month average SWC and the emissions the total cumulative gas emissions. (TIF) [file pone.0289859.s005.tif]
