# Supplementary material for: Earthworms and plants can decrease soil greenhouse gas emissions by modulating soil moisture fluctuations and soil macroporosity in a mesocosm experiment
Source: PLoS One. 2024 Feb 15;19(2):e0289859. doi: 10.1371/journal.pone.0289859 (PMC10868744; doi:10.1371/journal.pone.0289859)
Supplement: S1 Table — The “ns” abbreviation stands for variables that were not significant and were not retained in the minimal adequate models whereas mr2 represents the marginal coefficient of determination. ***P < 0.001; **P < 0.01; *P< 0.05; +P < 0.1. (DOCX) [file pone.0289859.s006.docx]

**Table S1.** Effects earthworms (Ew) and plant treatments on the total macroporosity volume (pores) as well as differentiated as burrows and cracks (see Table 2 for detailed variable description). The “ns” abbreviation stands for variables that were not significant and were not retained in the minimal adequate models whereas _m_r^2^ represents the marginal coefficient of determination. ***P < 0.001; **P < 0.01; *P< 0.05; ^+^P < 0.1.

| Source | Vpores_L1 | Vpores_L2 | Vpores_L3 | Vpores_L4 | Vpores_tot |
| --- | --- | --- | --- | --- | --- |
| Ew | ns | 9.04*** | 32.04*** | 191.71*** | 11.91*** |
| Plant | ns | 26.63*** | 21.36*** | 13.83*** | 15.78*** |
| Ew×Plant | ns | ns | ns | ns | ns |
| _m_r^2^ | 0.00 | 0.48 | 0.68 | 0.94 | 0.59 |
| Source | Vburrow_L1 | Vburrow_L2 | Vburrow_L3 | Vburrow_L4 | Vburrow_tot |
| Ew | ns | 42.87*** | 121.18*** | 258.36*** | 46.97*** |
| Plant | ns | 13.06*** | ns | ns | 7.85** |
| Ew×Plant | ns | ns | ns | ns | ns |
| _m_r^2^ | 0.00 | 0.72 | 0.98 | 0.99 | 0.99 |
| Source | Vcracks_L1 | Vcracks_L2 | Vcracks_L3 | Vcracks_L4 | Vcracks_tot |
| Ew | 3.83* | 17.56*** | 3.86* | 81.88*** | 5.95*** |
| Plant | ns | 16.95*** | 30.22*** | 23.25*** | 17.25*** |
| Ew×Plant | ns | ns | ns | ns | ns |
| _m_r^2^ | 0.18 | 0.49 | 0.43 | 0.63 | 0.40 |
