# Supplementary material for: Earthworms and plants can decrease soil greenhouse gas emissions by modulating soil moisture fluctuations and soil macroporosity in a mesocosm experiment
Source: PLoS One. 2024 Feb 15;19(2):e0289859. doi: 10.1371/journal.pone.0289859 (PMC10868744; doi:10.1371/journal.pone.0289859)
Supplement: Attachment — Submitted filename: Ganault et al. PlosONE - Reponse to reviewer.docx [file pone.0289859.s007.docx]

PONE-D-23-23009

Earthworms and plants can decrease soil greenhouse gases emissions by modulating soil moisture fluctuations and soil macroporosity in a mesocosm experiment

Dear Dr. Ganault,

Thank you for submitting your manuscript to PLOS ONE. After careful consideration, we feel that it has merit but does not fully meet PLOS ONE’s publication criteria as it currently stands. Therefore, we invite you to submit a revised version of the manuscript that addresses the points raised during the review process.

Additional Editor Comments (if provided):

Dear Author(s),
I write to you regarding the manuscript PONE-D-23-23009 entitled "Earthworms and plants can decrease soil greenhouse gases emissions by modulating soil moisture fluctuations and soil macroporosity in a mesocosm experiment" which you have submitted to PLOS One.
The reviewers have now commented on the manuscript. You will see that they are advising substantial revision of the manuscript. In addition to the reviewers’ comments, the overall write-up and the figures’ quality needs considerable improvement. When revising your work, please submit a list of changes or a rebuttal against each point being raised through track changes mode or by using bold or colored text.
Please revise the manuscript strictly according to the appended comments from the reviewers. While revising, need is to double check that all the references cited within the text have corresponding references.
Sincerely,

Reviewers' comments:

**Comments to the Author**

1. Is the manuscript technically sound, and does the data support the conclusions?
Reviewer #1: Yes
Reviewer #2: Partly

2. Has the statistical analysis been performed appropriately and rigorously?
Reviewer #1: Yes
Reviewer #2: I Don't Know

3. Have the authors made all data underlying the findings in their manuscript fully available?
Reviewer #1: Yes
Reviewer #2: Yes

4. Is the manuscript presented in an intelligible fashion and written in standard English?
Reviewer #1: Yes
Reviewer #2: Yes

5. Review Comments to the Author

**Reviewer #1**
Ganault et al. present data from a mesocosm experiment in which they investigated the combined effects of earthworms and plants on soil moisture, porosity, and CO2 and N2O emissions. The authors conclude that earthworms reduced greenhouse gas emissions via effects on soil porosity and thus moisture. The novelty of the study is the use of mesocosms in which both plants were present and earthworms could move more freely than in comparable laboratory experiments. The study is timely and written well. I provide some comments below on points that were unclear to me or could be improved.

Introduction
General: The intro is very much focused on N2O. I would like to see more information on how fluctuations in soil moisture, root exudates by plants, and earthworms can influence C stability and CO2 emissions from soils and on the mechanisms behind.
The introduction includes a specific paragraph on the effect of soil moisture on both N_2_O and CO_2_ emissions, exposing how water filled pore space determine gas and nutrient diffusion, limiting microbial activity when too low and too high values are reached. The mechanism is detailed first and more in length for N_2_O but is also valid for CO_2_ emissions.
The third paragraph of the introduction is dedicated to earthworm effect of carbon mineralization and stabilization.
We supplemented the paragraph on root exudates effects on CO_2_ in the beginning of the 4^th^ paragraph with additional references, as follow (L83-L86):
*“Root exudates contribute largely to carbon stabilization, more than plant litter, because the production of labile compounds (e.g. simple sugars) in the mineral soil favor the microbial driven formation of mineral associated organic matter [12,13,28]”*

L53/54 That earthworms increase CO2 and N2O emissions is far from being an established fact. I thus suggest using subjunctive here and/or citing other studies that found no changes in or reduced greenhouse-gas emissions in the presence of earthworms.
We agree with the reviewer, and that is why we performed our study. We changed the phrasing to subjunctive as recommended. However, the only existing meta-analysis on the topic suggest this increasing effect of earthworm on greenhouse gas emissions. We think that citing studies showing opposite results without citing the more abundant studies confirming this results would not be rigorous.

L56 ff Stabilization of C and/or N in soil is another important process affecting GHG emissions, which could/should be added here with appropriate references.
We reformulated the end of the sentence and added two references (Schmidt, et al. Nature. 2011;478: 49–56. doi:10.1038/nature10386; and Cotrufo et al. Glob Change Biol. 2013;19: 988–995. doi:10.1111/gcb.12113). It now reads L55-58:
*“However, the microbial processes causing greenhouse gas (GHG) emissions are extremely complex, and involve numerous interactions between earthworms, microbial communities, plants, and soil water and aeration status determining C and N mineralization or stabilization [9–13].”*

L59 delete "of"
There is no “of” in that line.

L63 Reference 13 is a case study and likely not sufficiently representative to support the statement made here. I suggest adding references.
We also added a reference reviewing our understanding of the general mechanisms behind this statement.

L97 ff In terms of the earthworm effects on water flows, it might be worth noting that anecics create mainly vertical burrows, while those of endogeics are mostly horizontal.
We detail this difference in burrowing activity and effect on water fluxes in the following (5^th^) paragraph of the introduction.

L124 Hypothesis 3) is not entirely clear. How do interactions between the ecological categories (if this is referred to here) affect GHG emissions?
This hypothesis is not on interactions between the ecological categories. Here we rather postulate that any difference in the effect between the two categories can be explained by the different burrowing patterns of those categories, which was never tested.

Methods
L162 Were these inputs comparable to those the soils receive under natural conditions?
These inputs represent 200 g.m^-2^ which can be achieved right after crop harvest in some systems. In our case, we also needed to ensure that enough food was provided to the earthworm, especially *L. terrestris*.

L163 This appears to be a wild mixture of litter inputs. Wouldn't it be more realistic to use litter from the species that was grown in the mesocosms? Why were these litter species chosen?
The crop where the soil originates is a rotation crop with maize, wheat, mustard and alfalfa so this organic residues are often found covering the soil there, while another plant species grows.

L170 Why was the earthworm biomass 2- to 3-fold higher in the mesocosms than under natural conditions?
On of our treatment consisted in *L. terrestris* alone, and having only one individual would have been to risky in case of mortality and have led to loose a replicate for one treatment.
We explained this better in the method section L184-188 as follows:
“For *L. terrestris*, adding the equivalent of the equivalent field biomass would mean introducing just one individual. Given the associated risk that the escape or death of this single individual could jeopardize the treatment, we chose to add two adult individuals of L. terrestris. As a result, the overall biomass is roughly double what is typically found per m^2 in agroecosystems.”

L176 How many replicates were there in total?
There is 7 replicates as already specified L191. We added the total number of mesocosms (n= 56) in L192.

L223 Not clear what the authors refer to here with "the two block".
Mesocosms were set on 2 tables in the greenhouse, one being in the south and the other north part of the greenhouse. We considered these as block, mesocosms position was randomized within those blocks and gas measurement occurred separately for each block.

L264 I wonder how this visual estimation looked like, how precise it can be, and what 5% intervals are. Maybe the authors could provide more info on this.
The same person (lead author) performed this measurement to avoid observer bias. Given the thin litter layer, it was easy to distinguish litter from bare soil and visually estimate the proportion or soil covered by litter, as recommended for example for canopy cover (see Anderson, E. W. (1986). A guide for estimating cover. Rangelands, 236-238.)

Results
L393 ff Again, how exactly was litter cover assessed? Was there a standard approach for this? I cannot imagine that simply looking at the litter can yield sound results.
This measurement sure is not as precise as image-based monitoring of soil cover but was the only method to monitor litter cover non destructively at the time of the experiment. The fact that this variable responded to the treatment is a proof that it provides a good proxy for litter burial by earthworm feeding activity.

L423 Better "model performance"
This was corrected, now L437, thank you.

Discussion
L448 Better "cumulative N2O emissions"
We think that both phrasing are correct and understandable and reads better when have to refer to both CO_2_ and N_2_O cumulative emissions.

L486 "burrows"; in general, the authors should recheck their manuscript for typos and grammar.
Checked, thank you.

L495 "root"; it is unclear why aggregate stability should be related to a reduced macropore volume; moreover, there is nothing about aggregates or pore volume in the cited reference.
We revised this section and changed the reference. It now reads L507-509:
*“In contrast, plants significantly reduced soil macropore volume, likely due to roots improving soil structure and stability against crack formation through the production of exudates acting as binding agent or by root mechanical engagement with soil aggregates [65,66].”*

L517 ff But the current study is short-term as well.
We revised this end of paragraph so the statement fits better our study and the recommendations made by Lubbers et al 2013. It now reads L531-534:
*“Overall, our study supports the idea that investigating the effect of earthworm on GHG emissions requires the use of experimental set-up that include plants, during long enough period (> 3months) [7], and allowing water availability to fluctuate due to biological activity.”*

L521 Do the authors have references for this "common practice"?
We removed this sentence.

L522 ff I appreciate the fact that the authors included a limitations paragraph in their manuscript. Points that could be added are that their experiment was relatively short-term and that earthworm densities strongly exceeded those commonly found in the field.
We added the following sentences (L562-565).
*“Finally, our study duration (3 month) was intermediate as classified by Lubbers et al 2013 and earthworm densities in two of our three earthworm treatment exhibited higher than natural densities. Future studies should perform longer experience with natural densities [7], which, as in our case, can prove to be technically challenging.”*

L549/550 In the text above, the authors state that measurement of emissions after watering events leads to bias. The statements here and above are somewhat contradictory.
Our original point was increased measurement frequency would provide more detailed and precise estimation of GHG emissions. However we do see the potential contradictory interpretation here and decided to remove this sentence as it was not absolutely necessary to understand our argument.

Fig. 4 All panels. I suggest not plotting trend lines for non-significant regressions. It is then easier for the reader to assess significant and non-significant results.
We removed confidence interval of non-significant relationships and we precised it in the figure caption.

Fig. S5. Not clear whether relations are significant or not.
The interaction is not significant (see Table 1). We added the correlation equations, and removed the confidence interval as is Fig 4 following the reviewer suggestion.

**Reviewer #2:**

The manuscript “Earthworms and plants can decrease soil greenhouse gases emissions by modulating soil moisture fluctuations and soil macroporosity in a mesocosm experiment” reports the results of greenhouse mesocosm experiment. The authors have shown that presence of earthworms in soil greatly reduced the emissions of greenhouse gases.
The quality of the figures is not good and needs to be improved. Legends cannot be read since in most figures they are blurred. The figures must be revised with better quality. Moreover, figure number and their caption are missing which makes it difficult to follow results and their interpretation.
The figure complied with PlosONE requirements and although they do appear in low quality on the pdf, they can be downloaded at high resolution. Figure number and caption is in the manuscript text.

English of the manuscript needs to be improved. It is suggested to authors to get proofread by native English speaker to get rid of small mistakes. For instance, in methodology (Line 133-136) and at many other places authors switch between past and present tense. It is advisable to present this section in past tense.
English was revised by one of the author with great publishing experience. Past tense was checked in the methods section.

It is difficult to follow the results of tomographic analyses. It is strongly suggested to clearly cite the figures as well as add figure captions.
We carefully checked that the figure were properly cited. The captions are available in the Supplemetary Material document.

6. PLOS authors have the option to publish the peer review history of their article ([what does this mean?](https://journals.plos.org/plosone/s/editorial-and-peer-review-process#loc-peer-review-history)). If published, this will include your full peer review and any attached files.

**Do you want your identity to be public for this peer review?** For information about this choice, including consent withdrawal, please see our [Privacy Policy](https://www.plos.org/privacy-policy).

Reviewer #1: No

Reviewer #2: **Yes:** Sohaib Aslam
