# Supplementary material for: Earthworms and plants can decrease soil greenhouse gas emissions by modulating soil moisture fluctuations and soil macroporosity in a mesocosm experiment
Source: PLoS One. 2024 Feb 15;19(2):e0289859. doi: 10.1371/journal.pone.0289859 (PMC10868744; doi:10.1371/journal.pone.0289859)
Supplement: Attachment — Submitted filename: Ganault et al. PlosONE - Reponse to reviewer.docx [file pone.0289859.s008.docx]

PONE-D-23-23009R1

Earthworms and plants can decrease soil greenhouse gases emissions by modulating soil moisture fluctuations and soil macroporosity in a mesocosm experiment

PLOS ONE

6. Review Comments to the Author

Please use the space provided to explain your answers to the questions above. You may also include additional comments for the author, including concerns about dual publication, research ethics, or publication ethics. (Please upload your review as an attachment if it exceeds 20,000 characters)

Reviewer #1: The authors mostly well addressed my comments. However, I have two further remarks:
1. I suggest using more suitable references at the end of the sentence in L55-58, as neither Cotrufo et al. nor Schmidt et al. specifically refer to how earthworms influence C stability or CO2 emissions. There are newer and more appropriate references for this statement, and I believe the authors are capable of finding and citing these references here. They may also amend this statement by referring to how earthworms influence aggregation, and thus stabilization of SOM and C, or other formation processes of stabilized C, which would better address my original concern.
This part of the paragraph is not only about how earthworms influence C stability or CO2 emissions but on the different biota and abiotic factors that determine greenhouse gases emissions. Still, we included one additional reference by Angst et al., Geoderma 289 (2017) 29–35 L58. Another paragraph, that also was revised to better address the original concern, is entirely dedicated to this topic lines 76-86.

2. Regarding the litter cover assessment: I suggest providing more information or a justification in the manuscript of why the authors think the method was sound
We rewrote the material and method on this topic and added two references to better justify our method.

Reviewer #2: Authors tried to address the comments, however, I still feel there is problem with English language.
We submitted our manuscript to the service American Journal Expert and provided the certificate of translation. The edits were made on the revised version of the manuscript without track changes and are therefore not visible on the main manuscript but was attached as a supplementary file.

7. PLOS authors have the option to publish the peer review history of their article ([what does this mean?](https://journals.plos.org/plosone/s/editorial-and-peer-review-process#loc-peer-review-history)). If published, this will include your full peer review and any attached files.


If you choose “no”, your identity will remain anonymous but your review may still be made public.


**Do you want your identity to be public for this peer review?** For information about this choice, including consent withdrawal, please see our [Privacy Policy](https://www.plos.org/privacy-policy).

Reviewer #1: No

Reviewer #2: No
